# Nullification in an Eclectic

**Published:** 1887-06

**Authors:** 


NULLIFICATION BY AN ECLECTIC.—In Alabama there is a law requiring practitioners to report statistics of deaths to a health officer. As the law was procured through the agency of the Regular profession, the Eclectics do not cheerfully comply, and, at a late meeting of the Eclectic Association, they resolved not to obey the Allopathic law for collecting statistics. We note that, in Lee County, the ball has opened, Dr. Lamar, Eclectic, of that county, having refused to report, received a communication from Dr. Atkinson, the county health officer, in which he says : " I am ordered by the County Board of Censors to compel you to report." To this Lamar replies tartly and defiantly. What will be the result we know not, but we suppose a legal process in which the constitutionality of the law will be tested.

